# A Review of Protocols for Fiducial Reference Measurements of Downwelling Irradiance for the Validation of Satellite Remote Sensing Data over Water

**DOI:** 10.3390/rs11151742

**Published:** 2019

**Authors:** Kevin G. Ruddick, Kenneth Voss, Andrew C. Banks, Emmanuel Boss, Alexandre Castagna, Robert Frouin, Martin Hieronymi, Cedric Jamet, B. Carol Johnson, Joel Kuusk, Zhongping Lee, Michael Ondrusek, Viktor Vabson, Riho Vendt

**Affiliations:** 1. Royal Belgian Institute of Natural Sciences (RBINS), Operational Directorate Natural Environment, 29 Rue Vautierstraat, 1000 Brussels, Belgium; 2. Physics Department, University of Miami, Coral Gables, FL 33124, USA; 3. Institute of Oceanography, Hellenic Centre for Marine Research (HCMR), Former US Base Gournes, 71500 Hersonissos, Crete, Greece; 4. University of Maine, Orono, ME 04469, USA; 5. Protistology and Aquatic Ecology Research Group, Gent University, Krijgslaan 281, 9000 Gent, Belgium; 6. Scripps Institution of Oceanography, University of California San Diego, 8810 Shellback Way, La Jolla, CA 92037, USA; 7. Institute of Coastal Research, Helmholtz-Zentrum Geesthacht (HZG), Max-Planck-Str. 1, 21502 Geesthacht, Germany; 8. Université du Littoral-Côte d’Opale, CNRS, Université de Lille, UMR8187, F62930 Wimereux, France; 9. National Institute of Standards and Technology (NIST), 100 Bureau Drive, Gaithersburg, MD 20899, USA; 10. Tartu Observatory, University of Tartu, 61602 Tõravere, Estonia; 11. School for the Environment, University of Massachusetts Boston, 100 Morrissey Blvd., Boston, MA 02125-3393, USA; 12. National Oceanic and Atmospheric Administration (NOAA), Center for Weather and Climate Prediction, 5830 University Research Court, College Park, MD 20740, USA

**Keywords:** downwelling irradiance, satellite validation, Fiducial Reference Measurements, water reflectance

## Abstract

This paper reviews the state of the art of protocols for the measurement of downwelling irradiance in the context of Fiducial Reference Measurements (FRM) of water reflectance for satellite validation. The measurement of water reflectance requires the measurement of water-leaving radiance and downwelling irradiance just above water. For the latter, there are four generic families of method, using: (1) an above-water upward-pointing irradiance sensor; (2) an above-water downward-pointing radiance sensor and a reflective plaque; (3) a Sun-pointing radiance sensor (sunphotometer); or (4) an underwater upward-pointing irradiance sensor deployed at different depths. Each method—except for the fourth, which is considered obsolete for the measurement of above-water downwelling irradiance—is described generically in the FRM context with reference to the measurement equation, documented implementations, and the intra-method diversity of deployment platform and practice. Ideal measurement conditions are stated, practical recommendations are provided on best practice, and guidelines for estimating the measurement uncertainty are provided for each protocol-related component of the measurement uncertainty budget. The state of the art for the measurement of downwelling irradiance is summarized, future perspectives are outlined, and key debates such as the use of reflectance plaques with calibrated or uncalibrated radiometers are presented. This review is based on the practice and studies of the aquatic optics community and the validation of water reflectance, but is also relevant to land radiation monitoring and the validation of satellite-derived land surface reflectance.

## 1. Introduction

The objective of this paper is to review the state-of-the-art of protocols for the measurement of downwelling irradiance, as used for the validation of satellite remote sensing data over water.

### 1.1. The Need for Fiducial Reference Measurements for Satellite Validation

Satellite remote sensing data is now used routinely for many applications, including the monitoring of oceanic phytoplankton in the context of global climate change, the detection of harmful algal blooms in coastal and inland waters, the management of sediment transport in coastal water, estuaries, and ports, the optimization and monitoring of dredging operations, etc. [1]. To be able to trust and use the remote sensing data, this must be validated, usually by a “matchup” comparison of simultaneous measurements by satellite and in situ. The terminology of “Fiducial Reference Measurements (FRM)” was introduced to establish the requirements on the in situ measurements that can be trusted for use in such validation. Using the definition proposed by [2] in the context of sea surface temperature measurements, the defining mandatory characteristics of a “Fiducial Reference Measurement (FRM)” are:

- An uncertainty budget for all FRM instruments and derived measurements is available and maintained, and is traceable where appropriate to the *International System of Units*/*Système International d’unités* (SI), ideally through a National Metrology Institute;
- FRM measurement protocols and community-wide management practices (measurement, processing, archive, documents, etc.) are defined and adhered to;
- FRM measurements have documented evidence of SI traceability that is validated by an intercomparison of instruments under operational-like conditions;
- FRM measurements are independent from the satellite retrieval process.

The second term above, given in bold, situates the current review, which should provide such a definition of measurement protocols for the downwelling irradiance measurement.

### 1.2. Scope and Definitions

**Figure 1.**
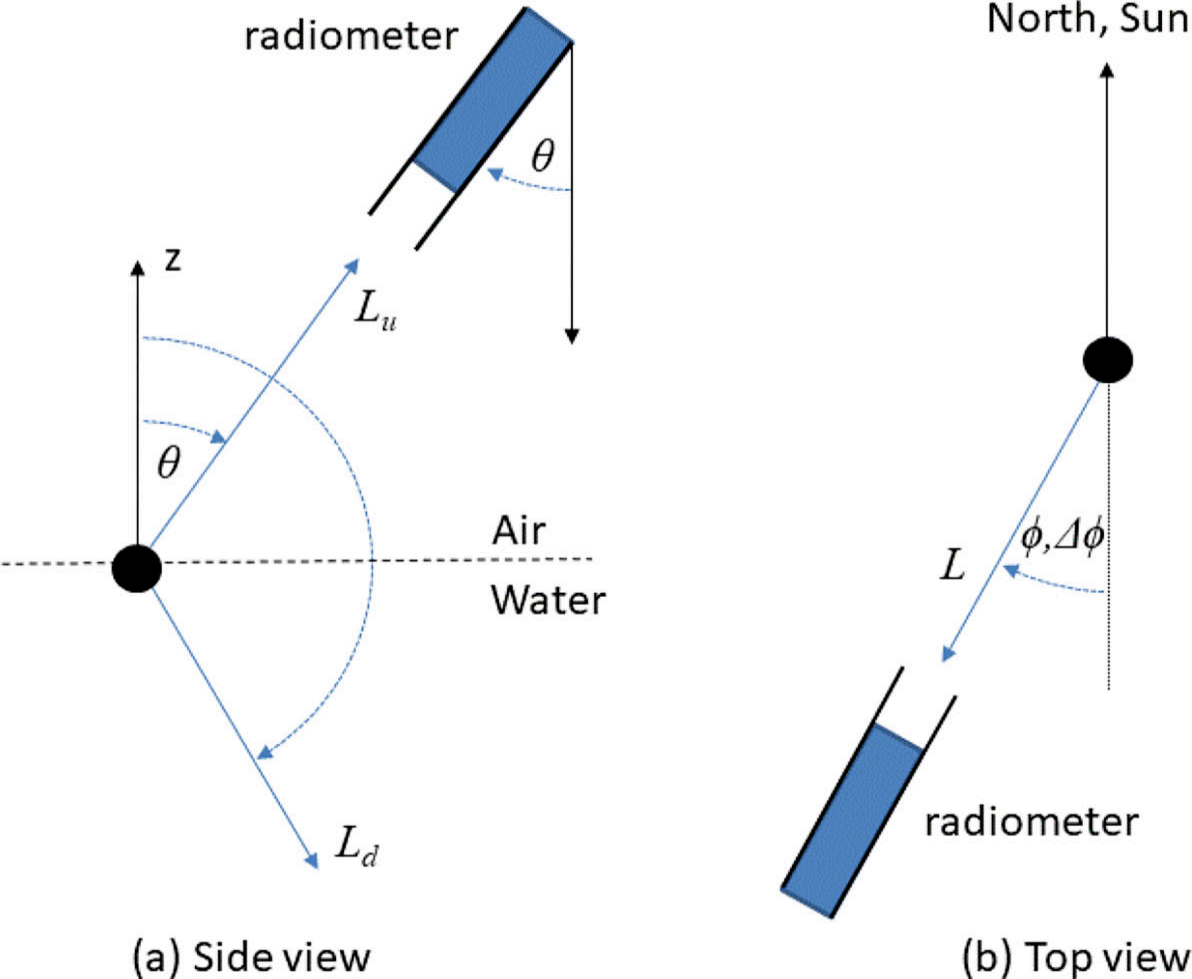
Nadir and azimuth viewing angle conventions illustrated for a reference system centered on the water surface (black dot). (**a**) Viewing nadir angle, θ, is measured from the downward vertical axis: upward radiances are viewed at θ<π/2, downward radiances (from sky and Sun) are viewed at θ>π/2. (**b**) Azimuth viewing angle, ϕ, and relative azimuth viewing angle, Δϕ, are measured for viewing directions clockwise from the north and Sun respectively: radiance viewed by a radiometer pointing toward north has an azimuth viewing angle of 0, and radiance viewed by a radiometer pointing toward and away from the Sun have relative azimuth viewing angles of 0 and π, respectively.

This review is focused on the validation of satellite data products for water reflectance at the bottom of the atmosphere. In the present review, the terminology of “remote sensing reflectance”, $R_{rs}$, is used as shown in Equation (1):

(1) $$R_{rs}(\lambda ,\theta ,\phi )=\frac{L_{w}(\lambda ,\theta ,\phi )}{E_{d}^{0+}(\lambda )}$$

where $E_{d}^{0+}(\lambda )$ is the above-water downwelling irradiance, which is also called the “spectral downward plane irradiance”, and $L_{w}(\lambda ,\theta ,\varphi )$ is the water-leaving radiance [3], after the removal of the air–water interface reflection, just above the water in the upward direction measured by the radiance sensor and defined by nadir viewing angle θ and azimuth angle φ. The conventions used for these angles are defined in Figure 1.

$E_{d}^{0+}(\lambda )$ is itself defined [3] as the integral of radiance, L(λ,θ,ϕ), over the downward hemisphere of solid angles (giving the geometric factor Sinθ) and weighted by |Cos(θ)| (since this is plane irradiance) and is measured in ${\text{Wm}}^{-2}\;{\text{nm}}^{-1}$:

(2) $$E_{d}^{0+}(\lambda )=\int_{\phi =0}^{2\pi }\int_{\theta =\pi /2}^{\pi }L(\lambda ,\theta ,\phi )|Cos(\theta )|Sin\theta d\theta d\phi$$

In the following text, λ, θ, and ϕ are omitted in the notations for brevity.

The θ integral limits from π/2 to π in Equation (2) correspond to the nadir viewing angle convention defined in Figure 1, but are different from the integration limits from 0 to π/2 found in some references, e.g., Equation (2.9) of [4], which defines θ as the incidence angle of photons from air. While there is diversity in the nadir/zenith angle terminology in different references, and Figures 2.1 and 2.4 of [4] are themselves quite ambiguous in the use of θ, in practice it is not difficult to follow a consistent angle convention. Similarly, for azimuth angles, these may be defined in some references for the light propagation direction or for the direction toward which the radiometer is pointing (or, in satellite metadata, for the azimuth of the satellite/Sun as seen from the ground location). These azimuth angle conventions can easily be understood and converted provided that they are well defined.

Thus, the validation of $R_{rs}$ is based on simultaneous measurement of two parameters: $E_{d}^{0+}$ and $L_{w}$. A companion paper [5] focuses on the measurement of $L_{w}(\lambda )$. The present review focuses on the measurement of $E_{d}^{0+}$, reviewing the state-of-the-art of measurement protocols in the FRM context, particularly as regards components of the measurement uncertainty budget relating to the measurement protocol.

In addition to the use of $E_{d}^{0+}$ to enable the validation of satellite-derived reflectance, $E_{d}^{0+}$ measurements can also be used to validate separately the $E_{d}^{0+}$ (or equivalently the atmospheric transmittance) calculated as an intermediate product in satellite data-processing chains.

In some references, $E_{d}^{0+}$ may be called “surface irradiance”—typically with notation $E_{s}$—or more ambiguously “reference irradiance”. The parameter is most completely described as “above-water spectral downward horizontal plane irradiance”.

$E_{d}^{0+}$ is composed of photons that reach the surface directly from the Sun (“direct irradiance”) and of photons that reach the surface from the sky after scattering in the atmosphere (“diffuse irradiance”). The latter may also include some photons that have interacted with the surrounding surface and subsequently been backscattered in the atmosphere—see page 12 of [6].

**Figure 2.**
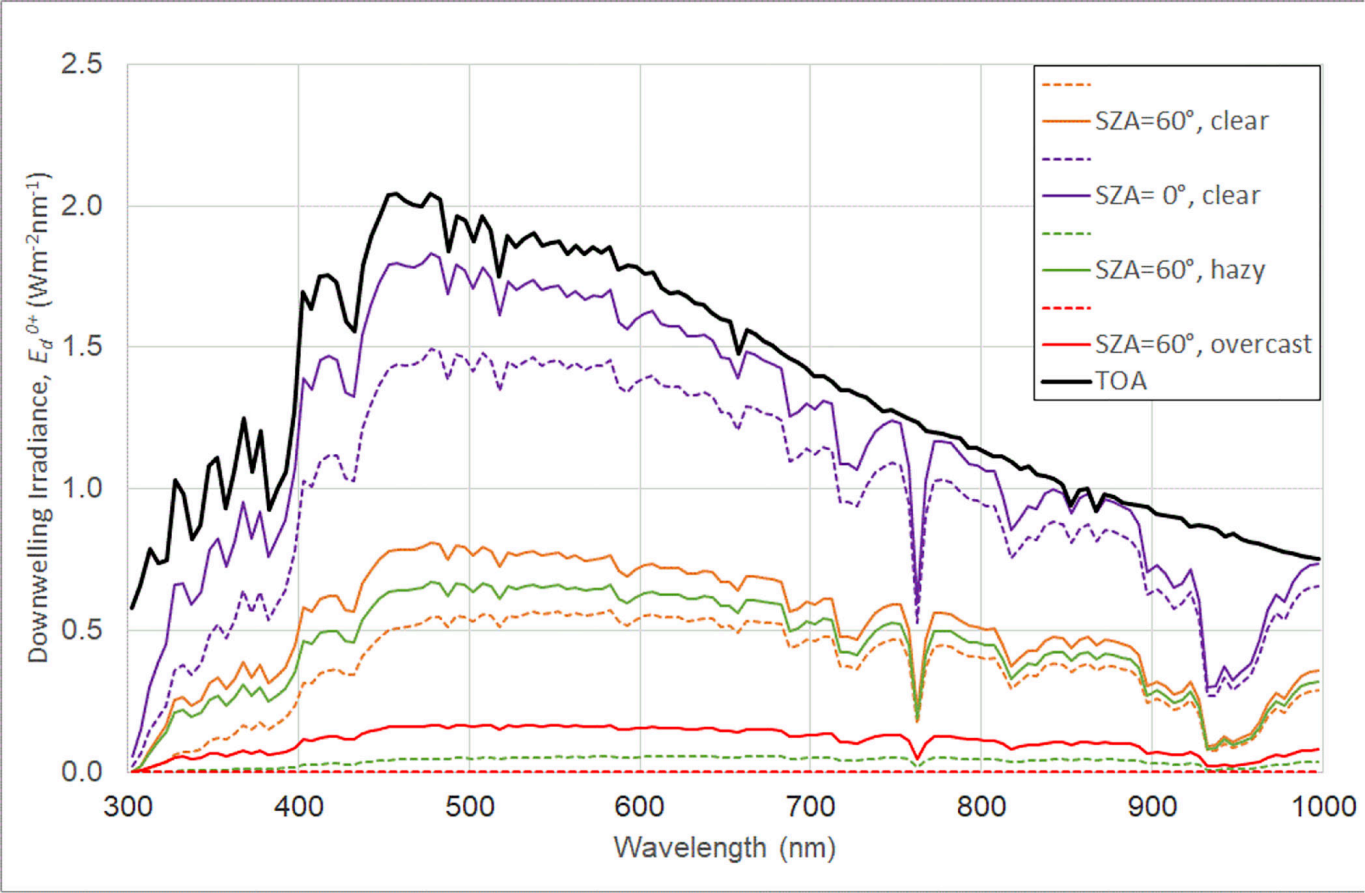
$E_{d}^{0+}$ for four combinations of Sun zenith angle (SZA) and atmospheric conditions, averaged over 5-nm bands. Solid colored lines are total $E_{d}^{0+}$; dashed lines are the corresponding direct component. The solid black line is the band-averaged extraterrestrial solar downwelling irradiance for comparison. Redrawn from [7].

Thus, $E_{d}^{0+}$ spectra are related to: (a) the extraterrestrial solar irradiance, (b) the Sun zenith angle, (c) atmospheric scattering and absorption from molecules, aerosols, and clouds, and (d) to a lesser extent, surface reflectance. Some typical $E_{d}^{0+}$ are plotted in Figure 2 for different Sun zenith angles and atmospheric conditions.

In sunny, low to moderate Sun zenith angle conditions where direct irradiance is greater than diffuse irradiance, $E_{d}^{0+}$ varies over the day approximately according to the cosine of the Sun zenith angle. This temporal variability is greatest just after sunrise and just before sunset. The time averaging of replicate $E_{d}^{0+}$ measurements can be simple mean averaging with reference to a central time if the total duration for replicates is short or can be normalized by the cosine of the Sun zenith angle before averaging.

The present paper is focused on aquatic applications, including the full range, size, and diversity of water bodies from deep oceans through coastal and estuarine waters to ports and inland lakes. The measurement of $E_{d}^{0+}$ is required also for the radiometric validation of surface reflectance over land—such applications are not the focus of the present paper, although there are in principle no major differences between the measurement of $E_{d}^{0+}$ over land and over water. Measurements of $E_{d}^{0+}$ without simultaneous $L_{w}$ are also relevant, outside the $R_{rs}$ validation context, for a variety of applications, including monitoring the Earth’s radiation budget for climate applications [8,9], ground-level ultraviolet radiation [10,11] for health-related and ecosystem-related applications, photosynthetically available radiation for biological applications [12,13], solar energy and building applications [14], etc. These applications are not specifically covered here, although many considerations of the measurement protocols described here are valid for all such applications.

Using the terminology of [15], the spectral ranges of primary interest here are the visible (380 nm to 760 nm) and near infrared (760 nm to 1400 nm) ranges. The considerations for measurement of $E_{d}^{0+}$ given here should be valid also for the near ultraviolet (300 nm to 400 nm) and middle infrared (1400 nm to 3000 nm), although the importance of the various uncertainty sources may be different because of the different intensity and angular distribution of downwelling irradiance, and the equipment (irradiance/radiance sensor, reflectance plaque) may have different properties in these ranges.

The protocols described here are relevant for the validation of a vast range of optical satellites, including the dedicated medium resolution “ocean color” missions, such as AQUA/MODIS, Sentinel-3/OLCI, NPP/VIIRS, etc., but also the operational high spatial resolution missions such as Landsat-8/OLI and Sentinel-2/MSI, as well any other optical mission from which water reflectance can be derived, including the geostationary COMS/GOCI-1 and MSG/SEVIRI, the extremely high resolution Pléiades and PlanetDove constellations, etc.

The current document does not try to identify a “best” protocol; it cannot provide typical uncertainty estimates if good practice is followed (that depends on many factors) and does not aim to prescribe mandatory requirements on specific aspects of a measurement protocol such as “acceptable tilt” or “minimum distance for ship shadow avoidance”. While such prescriptions have great value in encouraging convergence of methods and challenging scientists to make good measurements, the diversity of aquatic and atmospheric conditions where validation is required, the diversity of radiometers and platforms, and the corresponding diversity of measurement protocols suggests that more flexibility is needed. This flexibility is acceptable, provided that each measurement is accompanied by an SI-traceable uncertainty budget that is: (a) based on a full analysis of the protocol, and (b) that is itself validated, e.g., by measurement intercomparison exercises [16–18]. Then, the data user can accept or reject such measurements by applying their own threshold for “acceptable” measurement uncertainty.

The present review does aim to provide an overview of all the relevant protocols, including guidelines for radiometer deployment and the quality control of data and an overview of elements that should be considered in the complete uncertainty analysis of a measurement protocol. The approach is structured as follows: for each aspect of the measurement protocol contributing to measurement uncertainty, the perfect situation is summarized in a single sentence in boldface, e.g., “**the irradiance sensor should be vertical**”. This is followed by a discussion of techniques to achieve or monitor this (e.g., gimballing, measurement of tilt, removal of tilted data), practical considerations and problems (e.g., changes to ballasting of ships), and approaches to estimate uncertainty when this perfect situation is not achieved (e.g., model studies, experiments). While this highly structured approach may seem over-rigorous or even trivial (isn’t it obvious that an irradiance sensor should be vertical?), we do feel that it is necessary to be complete and rigorous in the FRM context (is it obvious to all measurement scientists that a reflectance plaque should be perfectly horizontal?).

For a general treatment of uncertainties in measurements, including a recommended terminology (e.g., “expanded uncertainty”) and generic methods for estimating each component uncertainty and combining uncertainties to achieve a total uncertainty, the reader is referred to the Guide to the Expression of Uncertainty in Measurement (GUM) [19].

The present review covers only aspects of the measurement relating to the protocol, including radiometer deployment, data acquisition, and processing aspects, but excluding any uncertainties arising from radiometer imperfections, such as calibration, thermal sensitivity, spectral response (straylight/out-of-band effects), non-linearity, and angular (cosine) response. These radiometer-related aspects deserve a review paper of their own; the reader is referred to Volume II of the NASA Ocean Optics Protocols [20], Section 3 of [21], Chapter 2 of [22] and to the papers in this volume, e.g., [23,24].

In the satellite validation context covered by this review, the focus is on clear sky conditions. There is no clear consensus regarding an objective definition of “clear sky” conditions, although Web Appendix 1 of [25] proposes for moderate Sun zenith angles the test $L_{d}/E_{d}^{0+}$ (750 *nm*) < 0.05, where $L_{d}$ is sky radiance at a 135° relative viewing azimuth to the Sun and a 140° viewing nadir angle. This test will detect clouds in front of the Sun because of the consequent increase in $1/E_{d}^{0+}$, and will detect clouds in the specified sky-viewing direction because clouds have greater $L_{d}$ values than blue sky at 750 nm. A more complete test for “clear sky” conditions could involve the use of hemispherical camera photos, but would need automated image analysis for an objective test.

### 1.3. Previous Protocol Reviews

Most of the pre-2004 in situ measurements of water reflectance were made for the purpose of oceanic applications, and most aquatic optics investigators base their measurement protocol in some way on the NASA Ocean Optics Protocols [20] and the references contained within that multi-volume publication. While there are no fully new methods for the measurement of $E_{d}^{0+}$ since the NASA 2004 protocols collection, the current review aims to better reflect the current practices. The main evolutions since 2004 include:

- more frequent use of unsupervised measurements for validation, e.g., AERONET-OC [26] and Bio-ARGO [27], instead of shipborne supervised measurements
- greater need for validation measurements in coastal and inland waters rather than the prior focus on oceanic waters
- preference for above-water measurement of $E_{d}^{0+}$ rather than extrapolation from underwater profiles
- reduction in the cost of radiometers facilitating use of an irradiance sensor (instead of a radiance sensor and a reflectance plaque), and better availability of hyperspectral radiometers.

### 1.4. Overview of Methods

Protocols for measurement of $E_{d}^{0+}$ are grouped into three broad families of method:

- Direct above-water measurement of $E_{d}^{0+}$ with an upward-pointing irradiance sensor (“Irradiance sensor method”)
- Estimation of $E_{d}^{0+}$ using a downward-pointing radiance sensor and a reflective plaque (“Reflectance plaque method”)
- Estimation of $E_{d}^{0+}$ from direct sunphotometry and a clear sky atmospheric model (“Sunphotometry method”)

A fourth family of method, estimating $E_{d}^{0+}$ from underwater measurements of downwelling irradiance at differences depths, $E_{d}(z)$, is now considered obsolete for measurement of $E_{d}^{0+}$—see Section 5.

For each family of method, the measurement equation is defined, and the measurement parameters are briefly described in Sections 2–4, respectively. The elements that should be included for the estimation of total protocol-related measurement uncertainty are discussed with some key considerations, guidelines, and recommendations. The “protocol-related” measurement uncertainty includes both known imperfections in the protocol (e.g., atmospheric models used in sunphotometry) and deployment-related imperfections (e.g., the tilting of sensors/plaques).

## 2. Direct above-Water Measurement of $E_{d}^{0+}$ with an Upward-Pointing Irradiance Sensor

### 2.1. Measurement Equation

Since $E_{d}^{0+}$ can be measured directly using radiometers that are designed to measure plane irradiance, the measurement equation here simply relates the electrical output of a radiometer to calibrated irradiance. Imperfections in such radiometers (angular response, spectral response, non-linearity, thermal sensitivity, etc.) contribute, of course, to the total uncertainty budget of the measurement, and the imperfect cosine response is an important consideration for the measurement of $E_{d}^{0+}$, e.g., [24,28].

**Figure 3.**
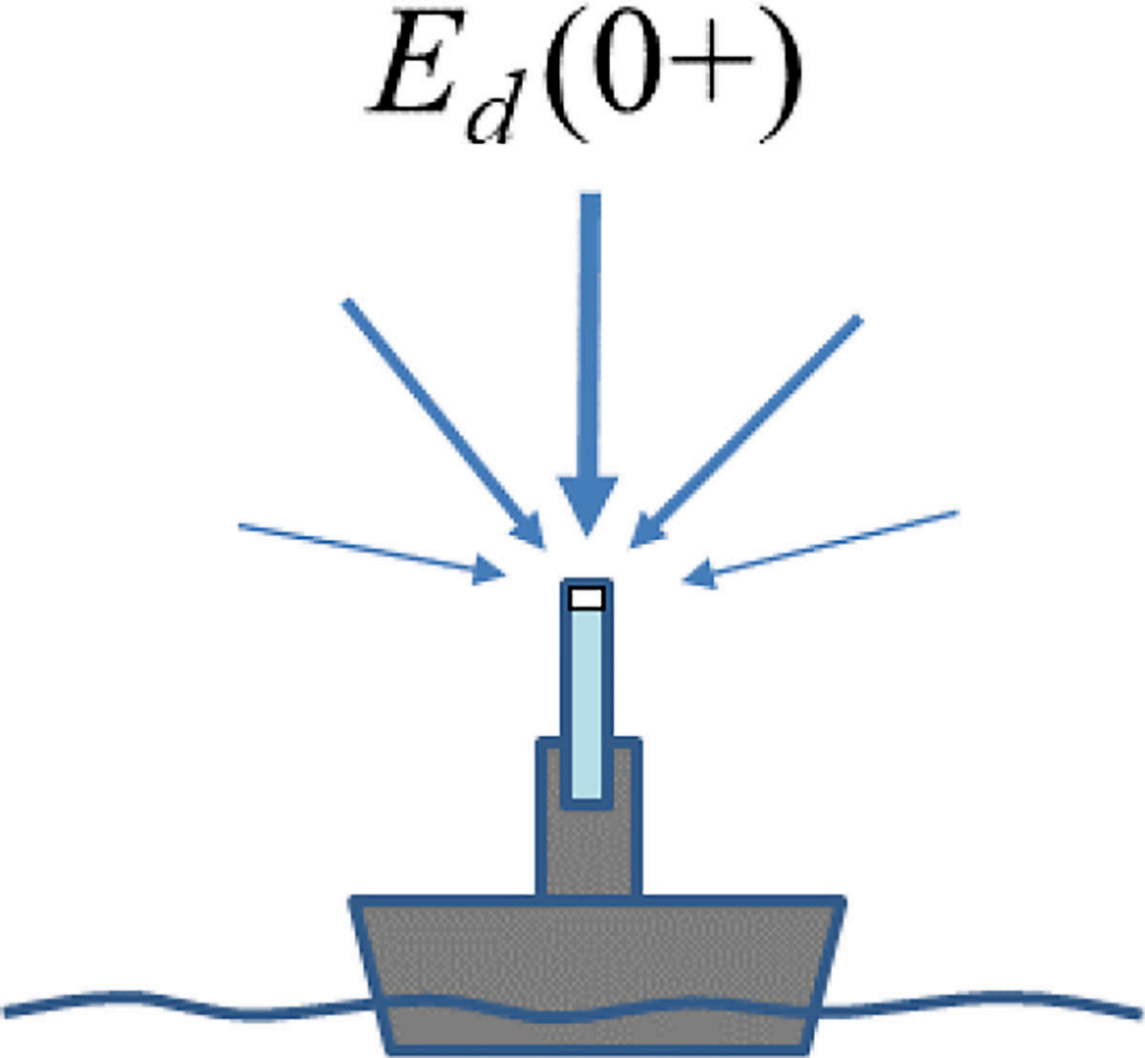
Schematic (not drawn to scale) of (shipborne) direct above-water measurement of $E_{d}^{0+}$ with an irradiance sensor (pale blue with flat white cosine collector).

The direct measurement of $E_{d}^{0+}$, which is sketched in Figure 3, can be made from various platforms including ships, small inflatable boats, buoys, fixed offshore structures, and underwater profiling platforms that contain a floating element or the ability to surface. These measurements can be either supervised or unsupervised. In all cases, it is recommended to mount the $E_{d}^{0+}$ radiometer as high as possible, above any superstructure elements and passing humans, in order to avoid the optical contamination of the measurement from the shading of both Sun and sky light. This can be achieved by the use of a fixed or telescopic mast, e.g., [29].

### 2.2. Protocol-Dependent Sources of Uncertainty

In addition to the radiometer-related sources of uncertainty that arise from imperfections in the radiometers themselves, including the angular (cosine) response of the radiometer, the direct measurement of above-water downwelling irradiance has a number of sources of uncertainty relating to the deployment conditions. These protocol-related sources of uncertainty are described in Sections 2.2.1–2.2.4.

#### 2.2.1. Tilt Effects

##### The irradiance sensor should be vertical.

The non-verticality of the $E_{d}^{0+}$ radiometer, e.g., caused by imprecise installation, wave-tilting of floating structures (buoys, ships), wind-tilting of offshore structures, including masts, and even ballast changes for ships (shifts in fuel, water, large equipment), will result in a bias in the measurement of $E_{d}^{0+}$. Therefore, it is necessary to measure the tilt of radiometers at sufficiently high frequency and perform the appropriate filtering of non-vertical data and/or averaging of data to reduce tilt effects.

For $E_{d}^{0+}$, the effect of tilt may be particularly strong in sunny (satellite validation) conditions because of the highly anisotropic light field. The main effect of tilt is similar to a change in the effective Sun zenith angle, and is strongest for tilt in the solar plane. The passive gimballing of an $E_{d}^{0+}$ sensor, if sufficiently well designed, may help to reduce tilt, as implemented in the DALEC system [30,31]. Active gimballing of an $E_{d}^{0+}$ sensor, using electric motors to correct for tilt, may now be feasible, although at the time of writing, the authors are not aware of documentation on the use of such hardware for $E_{d}^{0+}$ measurement.

The impact of tilt on measurement uncertainty can be estimated if the two angles of tilt with respect to the Sun are measured and the approximate angular variation of sky radiance is known, e.g., from imaging cameras, or estimated from atmospheric properties. At high tilt, an $E_{d}^{0+}$ sensor may also measure some light from the underlying water/land/platform surface instead of the sky, although grazing angle incident light has a low contribution to the cosine-weighted integral for $E_{d}^{0+}$.

Obviously, minimization of tilt can be a consideration in the design [32] or in the location (e.g., low waves) of validation measurement structures. Floating buoys and small ships may be particularly subject to high tilt.

#### 2.2.2. Shading from Superstructure

##### The irradiance sensor should be deployed above the height of all the other structures or objects (including humans).

The light field that is being measured may itself be perturbed by the presence of solid objects such as the superstructure of the platform used to mount them. This may be especially problematic on ships, where practical considerations may prevent mounting the $E_{d}^{0+}$ sensor above all other structures, particularly if regular inspection by humans of the fore-optics is required.

**Figure 4.**
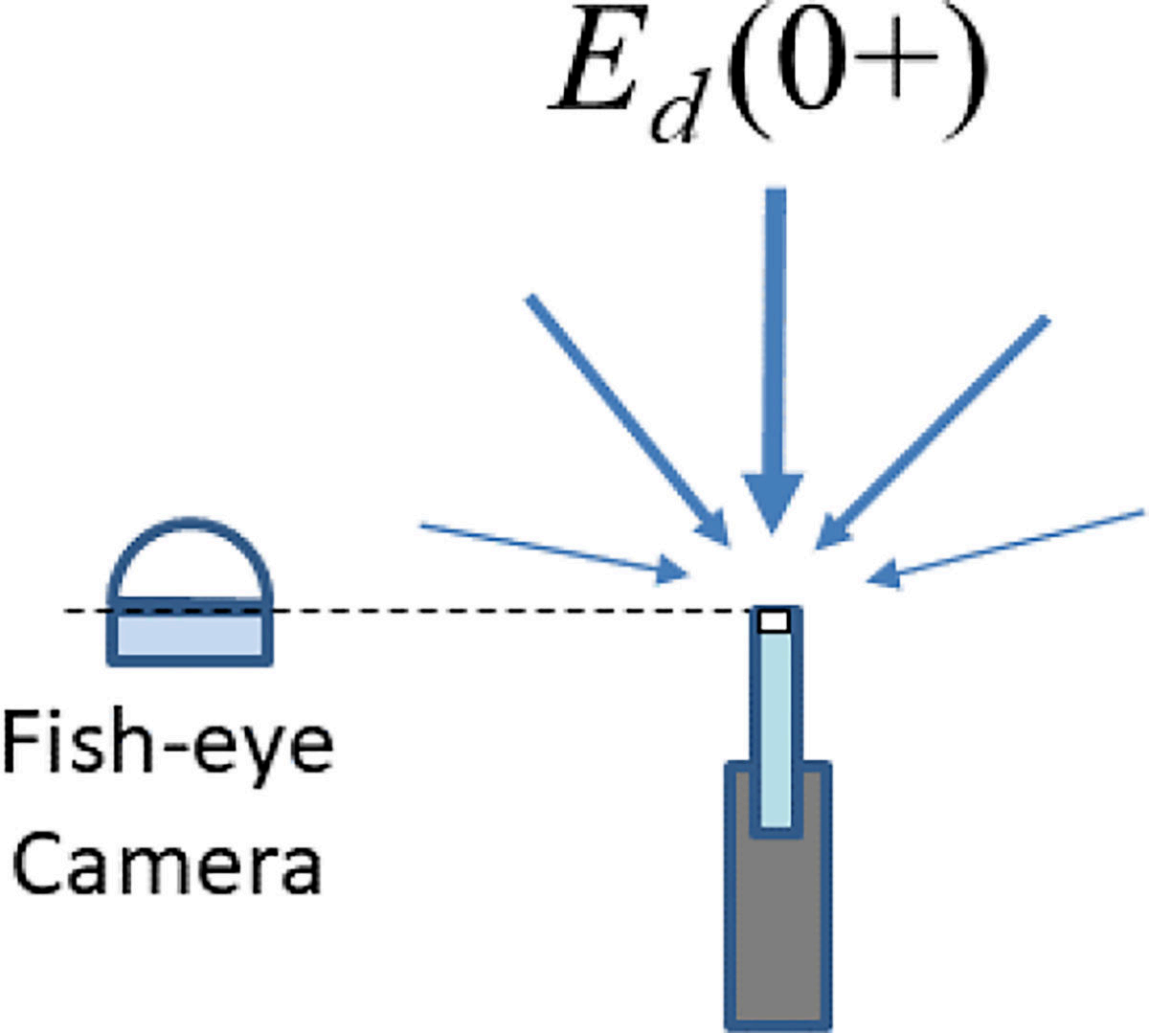
Schematic showing how a fish-eye camera, preferably fully hemispherical, can be used to qualitatively check for the superstructure contamination of $E_{d}^{0+}$ measurements.

**Figure 5.**
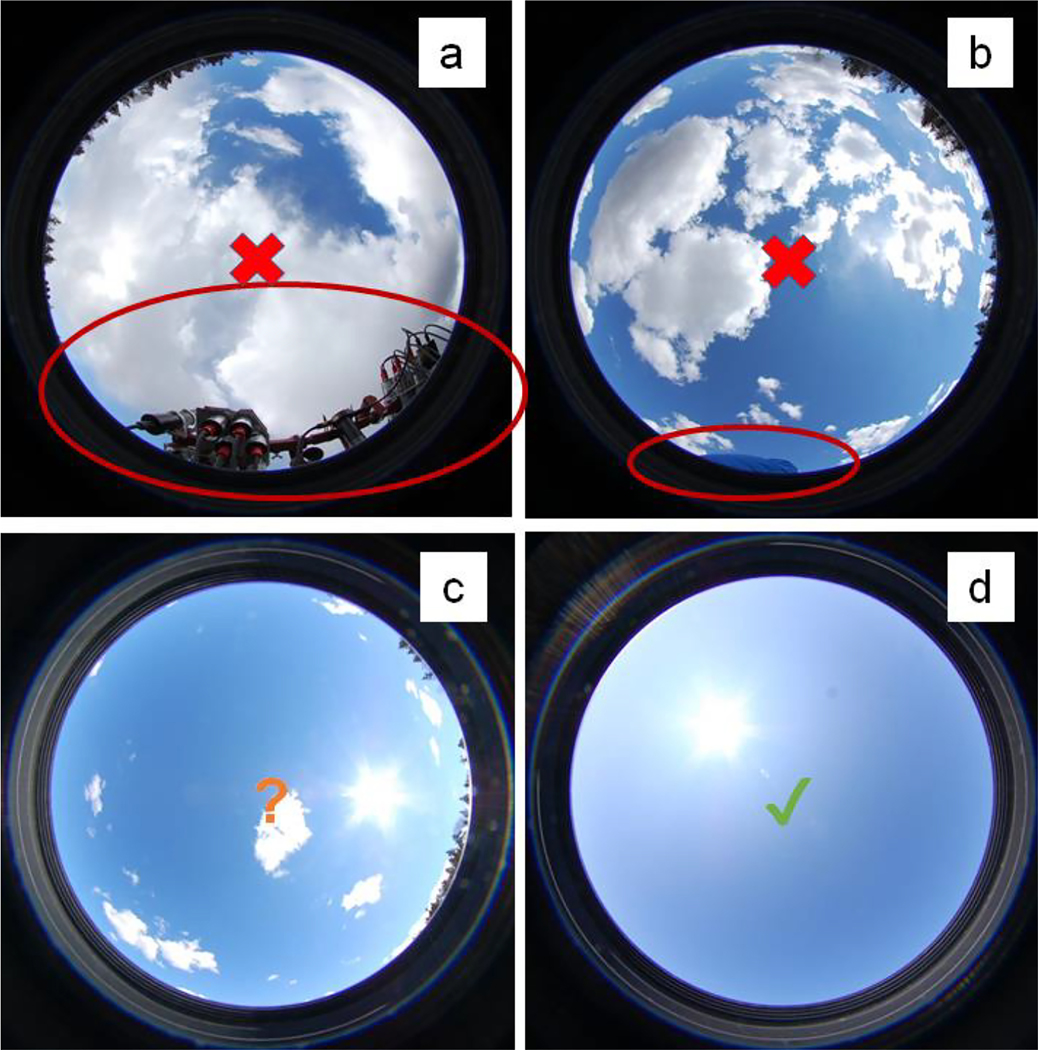
Example fish-eye photos taken to check for contamination of $E_{d}^{0+}$ measurements. (**a**) Contamination of field of view by other radiometers; (**b**) Contamination of field of view by a scientist in the bottom of the photo; (**c**) No contamination of field of view, partly cloudy sky; (**d**) No contamination of field of view, clear sky. The trees visible in the bottom-left photo, typical of inland water or very nearshore measurements, do affect the measurement, but are not considered as “contamination” in the context of this review. The impact of such far-field objects contributes to the natural downwelling irradiance at the measurement location, and should be measured as such.

The process of sky shading can be easily understood from fish-eye photographs taken vertically upwards at the location of an $E_{d}^{0+}$ sensor, as illustrated in Figures 4 and 5. Any part of the upward hemisphere that is not sky represents optical contamination of the measurement, and this contamination will be related to the solid angle of sky that is replaced by the object with near-zenith objects contributing more than near-horizontal objects to the cosine integral of radiances. Of course, it is best to make such photos with a calibrated fully hemispherical sky radiance camera [33]. However, even photos from simple cameras with a wide-angle lens and without any radiometric calibration can rapidly identify a major contamination of measurements from superstructures and/or other objects.

While direct Sun shadowing of the $E_{d}^{0+}$ sensor is generally avoided by design of the deployment method and can easily be identified and removed from data, the impact of more subtle optical contaminations of sky radiance can be more difficult to identify and estimate.

It is obvious that humans should remain fully below the level of an $E_{d}^{0+}$ sensor at all times during measurements. It is not unknown for resting birds to contaminate unsupervised $E_{d}^{0+}$ measurements [34], and measures may be taken to avoid this, e.g., the use of spikes below the field of view, but sufficiently close to threaten discomfort. Unusual contaminations may be identified by time series analysis or video camera monitoring of unsupervised installations.

On some platforms, optical contamination may also arise from atmospheric steam or smoke emissions from ship engine funnels and other exhaust gases (air conditioning, etc.).

Fixed offshore structures with limited access (e.g., oil and gas platforms, wind farm structures, navigational structures) as well as large ships with tall masts may be particularly subject to superstructure shading. Improvements in the stability of telescopic masts [35], which allow high mounting but easy inspection of fore-optics, and reductions in the price of such equipment should facilitate the adoption of deployment techniques with greatly reduced or zero superstructure shading.

For supervised shipborne $E_{d}^{0+}$ measurements, the use of a floating platform to carry the $E_{d}^{0+}$ radiometer away from the ship will clearly minimize—to possibly a negligible amount—the superstructure-related perturbations. This may be conveniently combined in a floating/profiling platform used for underwater profiling of upwelling radiance.

Measures to reduce and/or estimate the uncertainties associated with superstructure shading may include redundant measurements by multiple sensors located in different positions, and hence subject to different shading effects, or experiments with sensors at different heights/locations, etc. Three-dimensional (3D) radiative transfer modeling may also be used to estimate uncertainties in $E_{d}^{0+}$ measurements associated with superstructure effects.

#### 2.2.3. Fouling

##### The fore-optics of the irradiance sensor should be kept clean.

Upward-facing sensors needed for measuring $E_{d}^{0+}$ are prone to fouling of the fore-optics, especially during long-term unsupervised deployments.

Fouling may occur because of sea spray, the atmospheric deposition of particles (which may even embed within the structure of some diffuser materials used as fore-optics [36]), rain droplets, bird feces, etc. This can be mitigated by cleaning the fore-optics, and can be monitored by frequent calibration checks, e.g., with portable relative calibration devices [37].

Fouling is generally kept negligible for supervised deployments by regular inspection and, when necessary, the cleaning of fore-optics and protection by lens caps when not measuring (e.g., at night and between “stations” for discrete measurements).

Exposure to ultraviolet light can lead to the photodegradation of materials used as diffusers.

For unsupervised deployments, fouling and photodegradation can be minimized by the protection of fore-optics when not measuring by the use of external mechanical shutters [38] or the rotation of sensors to point downwards (typified by the “parking” function of the CIMEL CE-318 sunphotometer when not measuring).

Major fouling events can be identified by time series analysis of data and/or video camera imagery.

The uncertainty related to fouling can be estimated by comparing post-deployment calibrations before and after cleaning, although it is also noted that fouling may vary non-monotonically in time because of the cleaning effect of rain water. To separate the effects of fouling from intrinsic sensitivity changes (e.g., long-term drift or short-term changes typically caused by mechanical shock), these measurements must be done immediately before and after cleaning, e.g., in the field (using a stable light source such as a clear sky) or in a calibration laboratory (which must be provided with the uncleaned radiometer).

#### 2.2.4. Fast Natural Fluctuations

##### Measurements should be used only during periods of stable illumination.

In clear sky conditions, the natural variability of $E_{d}^{0+}$ over a typical measurement time scale (~1 to 10 min) is low, and may be easily estimated from a clear sky irradiance model, e.g., [39], using as input the temporal variation of the Sun zenith angle and an estimation/measurement of aerosol optical thickness.

If measurements are made during partially cloudy conditions, in addition to the tilt-induced fluctuations described in Section 2.2.1, the natural variability of $E_{d}^{0+}$ may be non-negligible, particularly if there are clouds or haze near the Sun. In such cases, careful quality control of data is necessary to remove individual measurements or complete sets of measurements that cannot be used for satellite validation. Quality control will typically include tests on temporal variability including second derivative “spike/jump” analysis and min/max/standard deviation analysis, and may also include the comparison of data with a clear sky model.

A full sky imager can be used to provide detailed information on sky conditions for quality control [40].

It is suggested here that FRM for satellite validation should not be made during fully cloudy conditions or when the Sun is obscured by clouds or haze. In situ measurements can be made at a slightly different times from the satellite overpass, e.g., 1 to 6 h depending on natural variability, and so a cloud-free satellite image could theoretically correspond with an in situ reflectance measurement made during cloudy conditions within an acceptable time window. However, many factors, including the very different bidirectional reflectance of water under a sunny or a cloudy sky, suggest that this should be avoided in the FRM satellite validation context. In other contexts, such as the simultaneous measurement of reflectance and chlorophyll *a* for algorithm calibration/validation, it may be acceptable to use measurements made in cloudy conditions, particularly fully overcast conditions, provided that the corresponding measurement uncertainties are sufficiently quantified and limited.

The question of whether FRM can be made in partially cloudy conditions is relevant. It can be argued that only the best measurements should be used, and this requires perfectly clear sky conditions. On the other hand, if a measurement scientist is able to estimate the uncertainties associated with partially cloudy conditions, then the data user could later decide whether to use or reject such measurements for their specific application on the basis of a threshold on measurement uncertainty. There is no clear consensus on this question at present, but perhaps the debate requires first a more objective definition of “cloudiness“ and/or “clear sky“ conditions—see Section 1.2. Isolated clouds with small solid angles, away from the Sun and low on the horizon, so with low zenith cosine weighting, have little impact on $E_{d}^{0+}$.

Uncertainties associated with fast natural fluctuations can be estimated from the standard deviation of replicate measurements made over a certain interval of time. High uncertainty may lead to simple rejection of the measurement.

### 2.3. Variants on the Method of Direct above-Water Measurement of $E_{d}^{0+}$ with an Upward-Pointing Irradiance Sensor

Underwater drifting floats used for satellite radiometry validation [27] may lack a permanently above-water $E_{d}^{0+}$ sensor, and make only occasional $E_{d}^{0+}$ measurements when surfacing. There is no fundamental difference between the “surfacing” $E_{d}^{0+}$ sensor and the permanently above-water $E_{d}^{0+}$ sensors considered in the rest of this review. However, it is noted that there may be different designs of $E_{d}^{0+}$ sensors for in-water and in-air measurements; the time and horizontal space differences between $E_{d}^{0+}$ and $L_{w}$ measurements must be considered; and the presence of water, as already mentioned in Section 2.2.3, and aquatic algae on the fore-optics may be more problematic.

With an additional moving “shadowband” accessory, it is possible to combine full Sun and sky $E_{d}^{0+}$ with a direct Sun-obscured measurement, thus giving the diffuse sky component of $E_{d}^{0+}$, which is termed $E_{d}$. This is not commonly used for the validation of satellite data over water, since the primary radiometric product from satellites, e.g., the reflectance product given in Equation (1), does not require a decomposition of $E_{d}^{0+}$ into direct and diffuse components. However, this additional information does provide the additional opportunity to validate the satellite data processing for direct and diffuse atmospheric transmittance, and does potentially allow improving the bidirectional reflectance distribution functions (BRDF) corrections. The measurement of direct and diffuse components of $E_{d}^{0+}$ can also be used to improve self-shading corrections when making underwater measurements of upwelling radiance. The measurement of $E_{d}^{dif}$ in addition to the total $E_{d}^{0+}$ is of major importance for other applications such as earth radiation budget monitoring, agriculture, solar energy, etc. A discussion of $E_{d}^{dif}$ data acquisition and processing with the shadowband technique can be found in [41].

## 3. Estimation of $E_{d}^{0+}$ Using a Downward-Pointing Radiance Sensor and a Reflective Plaque

### 3.1. Measurement Equation

**Figure 6.**
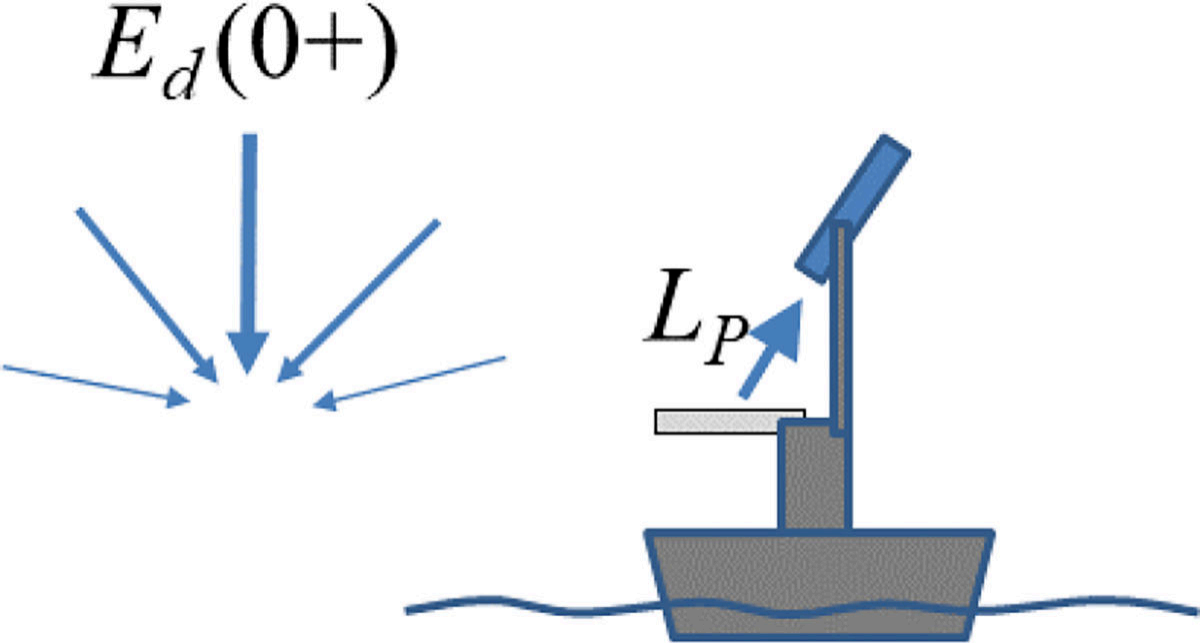
Schematic showing indirect measurement of $E_{d}^{0+}$ using a downward-pointing radiance sensor and a reflective plaque (sensor, plaque, and holder not to scale).

^$E_{d}^{0+}$^ can also be calculated indirectly by measuring the exitant radiance, $L_{P}$, from a horizontally deployed reflectance plaque of known reflectance, $\rho_{p}$—see Figure 6. If the plaque is perfectly Lambertian, then:

(3) $$E_{d}^{0+}=\frac{\pi *L_{p}}{\rho_{p}}$$

where all the terms may vary with wavelength, but the wavelength variation is dropped for brevity throughout this section. If the plaque is not perfectly Lambertian, then the downwelling light field can be approximated as a collimated beam of light from the Sun direction [42], giving the measurement equation:

(4) $$E_{d}^{0+}=\frac{L_{P}\left(\theta_{v},\phi_{v}\right)}{f_{r}\left(\theta_{i},\phi_{i},\theta_{v},\phi_{v}\right)}$$

where $f_{r}\left(\theta_{i},\phi_{i},\theta_{v},\phi_{v}\right)$ is the plaque bidirectional reflectance distribution function (BRDF), $\theta_{v}$, $\phi_{v}$ are the viewing nadir and azimuth angles and $\theta_{i}$, $\phi_{i}$ are the zenith and azimuth angles of the incident collimated beam, which are generally assumed to correspond to the Sun beam direction.

A common material for such plaques is sintered polytetrafluorethylene (PTFE), which is typically sold under the product name Spectralon^™^ (see disclaimer at the end before the references), which can be manufactured to give near 100% reflectance $\left(\rho_{P}\approx 1.0\right)$ for “white” plaques with low spectral variation of reflectance, low departure from the perfect Lambertian angular response [43], low spatial heterogeneity, and reasonable temporal stability. Lower reflectance “grey” plaques, e.g., $\rho_{P}\approx 0.18$, can also be used, although they have less Lambertian angular response. Other diffusive materials have been used in this method, including grey “cards” that are used traditionally in photography. All the materials used in the FRM context need to be adequately characterized as regards bidirectional, spectral, spatial and temporal variability.

Historically, the measurement of $E_{d}^{0+}$ using a downward-pointing radiance sensor and a Lambertian reflective plaque was adopted for cost considerations, allowing all the measurements to be made with a single radiance sensor. This method also allows the reduction of some calibration-related uncertainties, since only one sensor is used. Moreover, if only $R_{rs}$ is required, this method may be implemented with an uncalibrated sensor (but see the discussion in Section 3.1.1).

The reflectance plaque method is popular in the land remote sensing community, possibly because the measurements for some middle infrared wavelengths (1.4 μm to 2.5 μm) are important, which very significantly raises the cost of a radiometer and increases the uncertainty relating to cosine response for an irradiance sensor with a transmissive diffuser.

Measurements with a reflective plaque are often supervised, although it is possible to automate such measurements, e.g., [44].

Outside the FRM satellite validation context, the educational value of measurements made using this protocol, e.g., with very simple and inexpensive optical radiometers [45], is clearly recognized.

#### 3.1.1. Is It Necessary to Use a Calibrated Radiance Sensor?

The preparation of this review generated much discussion within the community regarding the question of whether an uncalibrated radiance sensor can be used to acquire measurements for satellite validation. This method was suggested in the NASA Ocean Optics protocols 2003 version “Method 2” [46] as being appropriate for the measurement of reflectance using an uncalibrated sensor. Indeed $R_{rs}$ can be calculated via Equation (1) from measurements of $L_{w}$ and $E_{d}^{0+}$ made by the same radiance sensor, even if this sensor is not calibrated, i.e., providing data for $L_{w}$ and $E_{d}^{0+}$ in (dark-corrected) digital counts rather than in SI-traceable units. While it is essential to characterize the sensor, e.g., for straylight, non-linearity, thermal effects, etc., it is not necessary to calibrate the sensor to perform radiometer-related corrections and uncertainty estimates. In fact, some radiometer-related uncertainties are best treated before calibration, e.g., non-linear effects may depend directly on the digital count data [47,48] (as compared to the maximum possible, saturated, digital counts), but not on the calibrated radiance.

There is formally nothing in the FRM definition that would require a calibrated radiance sensor to be used for the measurement of $R_{rs}$. However, the use of a calibrated radiance sensor does have two advantages:

- A calibrated radiance sensor will provide a calibrated $E_{d}^{0+}$, which can then be compared with clear sky models [39] for quality control purposes, and can be compared to satellite data to validate the computations of atmospheric transmittance (in addition to the more important $R_{rs}$ products).
- The interpretation of in situ measurement intercomparison exercises [17], as required by the FRM process, necessitates a separation of uncertainties arising from $L_{w}$ and $E_{d}^{0+}$ measurements, e.g., comparing $E_{d}^{0+}$ measurements from a vertically-mounted irradiance sensor (impacted by cosine angle uncertainties, etc.) with $E_{d}^{0+}$ measurements deduced from a radiance sensor viewing a reflectance plaque (impacted by BRDF uncertainties, etc.).

Moreover, it is noted [42] that the simple cancellation of unknown calibration factors used to calculate $R_{rs}=\pi L_{w}/E_{d}^{0+}$ in native spectral resolution no longer works precisely when spectrally convolving $L_{w}$ and $E_{d}^{0+}$ with a spectral response function, as needed for the validation of $R_{rs}$ for individual spectral bands of satellite sensors.

#### 3.1.2. What Nadir Angle Should Be Used for Viewing a Reflectance Plaque?

The NASA 2003 protocols (Volume III, Section 3.3) recommended that measurements of $E_{d}^{0+}$ with a reflective plaque should be made with a vertical downward (nadir) pointing radiance sensor and a plaque with BRDF calibration for varying downwelling light distributions (typically characterized by Sun zenith angle) and vertical upwelling reflected radiance. However, off-nadir viewing with the same nadir angle as water-viewing $L_{w}$ measurements, typically 40°, has often been adopted for practical reasons, e.g., for easy switching between plaque and water-viewing modes for certain deployments. It is noted that [49] provides the scientific basis for a water-viewing nadir angle of 40° (and relative azimuth to Sun of 135°) as a good geometry for sunglint avoidance, but does not give a scientific basis for a plaque-viewing nadir angle of 40°—the latter is merely suggested as practically convenient. On the other hand, an off-nadir plaque-viewing geometry may indeed be desirable for scientific reasons, since the radiometer shading of the plaque will be greater with nadir-viewing when the Sun zenith angle is low [42]. For off-nadir plaque viewing, there seems to be no standardization of the viewing azimuth angle, although the same azimuth angle as used for $L_{w}$ measurements (90° or 135° with respect to the Sun) would be a typical choice for both practical and shadow-avoidance reasons.

Optimal plaque-viewing geometry was investigated in [42], who recommend, for moderate Sun zenith angles between 20–60°, a plaque-viewing nadir angle of 40° for a ~100% reflective white plaque, to minimize operator/radiometer shading/reflection, but a nadir view for less reflective, grey plaques, where reflectivity may vary strongly with the viewing nadir angle. For both types of plaque, a viewing azimuth angle of 90° with respect to the Sun was recommended.

The FRM context does not prescribe a single viewing geometry (or any other specific aspect of a measurement protocol), but “simply” requires that, for whatever plaque-viewing geometry is adopted, the related uncertainties (radiometer and superstructure shading of plaque, plaque BRDF) be quantified.

### 3.2. Protocol-Dependent Sources of Uncertainty

In addition to the radiometer-related sources of uncertainty that arise from imperfections in the radiometers themselves, the measurement of $E_{d}^{0+}$ using a reflectance plaque has a number of sources of uncertainty relating to the deployment conditions. These protocol-related sources of uncertainty are described in Sections 3.2.1–3.2.7.

#### 3.2.1. Plaque Calibration

##### The reflectance plaque must be calibrated.

Clearly, the reflectance of the plaque used for this measurement must be calibrated with traceability to an SI standard and an uncertainty associated with this calibration. Optical contamination/degradation of the plaque and bidirectional effects are further considered in Sections 3.2.5 and 3.2.7.

#### 3.2.2. Plaque Homogeneity and Sensor Field of View

##### The reflectance plaque should be homogeneous and should fill the radiance sensor field of view.

It is known that plaques do have spatial and azimuthal inhomogeneities, and so it is assumed that the measurement area on the plaque corresponds sufficiently well to the area on the plaque used during plaque calibration, taking account of the surface average of any inhomogeneities.

Clearly, the plaque must fully fill, and preferably exceed, the sensor field of view (FOV) so that the measurement of $E_{d}^{0+}$ will not be contaminated by the background around the reflectance plaque. This can be facilitated by small FOV radiometers. In any case, the angular response of the radiance sensor should be checked for any residual response outside the manufacturer-specified FOV, e.g., by occulting the plaque partially with a black material moved from each edge of the plaque towards the center until an impact is detected

Uncertainties associated with the sensor field of view and plaque inhomogeneity can be assessed by experiments deploying the radiometers at different heights and at different horizontal locations above the reflectance plaque, and by changing the background around the reflectance plaque (since the radiometer shading effects will also vary with radiometer height—see Section 3.2.4).

#### 3.2.3. Tilt Effects

##### The reflectance plaque should be horizontal.

The non-horizontality of the reflectance plaque that is used for measurements of $E_{d}^{0+}$ will give uncertainty in the measurement of $E_{d}^{0+}$ in the same way as the non-verticality of an irradiance sensor used to directly measure $E_{d}^{0+}$, as discussed previously in Section 2.2.1. Tilting of the plaque can be caused by a number of factors, including imprecise leveling and, if measuring from a ship, ship roll during measurements. Therefore, it is necessary to measure the tilt of the plaque (not just the ship) at sufficiently high frequency and perform the appropriate filtering of non-horizontal data and/or averaging of data to reduce tilt effects.

Although digital inclinometers are now readily available for integration with radiometric data streams, they seem to not yet be used for shipborne measurement of $E_{d}^{0+}$ using a reflectance plaque.

For $E_{d}^{0+}$, the effect of tilt may be particularly strong in sunny (satellite validation) conditions because of the highly anisotropic light field, and the effect of a non-horizontal plaque is similar to a change in Sun zenith angle, and is strongest for tilt in the solar plane. At high tilt, the measurement may also measure some light from the water/land/platform instead of the sky, although the grazing angle incident light has a low contribution to the cosine-weighted integral for $E_{d}^{0+}$.

The impact of tilt on measurement uncertainty can be estimated if the two angles of tilt with respect to the Sun and approximate angular variation of the sky radiance (from imaging cameras or estimated from atmospheric properties) are known—see Section 2.2.1.

The minimization of tilt should be a consideration in the choice of measurement platform, taking account of expected wave conditions. Small ships may be particularly subject to high tilt because of larger ship roll.

#### 3.2.4. Shading from Superstructure and Radiometers and Mounting Equipment

##### The reflectance plaque should be deployed above the height of all other structures or objects (including humans).

The light field that is being measured is itself perturbed by the presence of solid objects anywhere above the level of the reflectance plaque. This includes, necessarily, the radiometer itself, which is used for measurements, but also any superstructure elements of the ship/platform as well as any equipment related to fixing the radiometer above the reflectance plaque.

The shading problems associated with this method are conceptually similar to those already described for direct measurement of $E_{d}^{0+}$ (Section 2.2.2), but are significantly worse:

- Firstly, there will always be some shading of sky radiance onto the plaque from the radiometer itself. The radiometer must be held above the plaque at a height that is sufficiently small so that the plaque fills the whole field of view of the radiometer. The exact height depends on the radiometer and the size of the plaque. Shading from the radiometer (and any associated fixations) will be related to the zenith cosine-weighted solid angle of sky filled by the radiometer, as seen from any point on the reflectance plaque, and will be worse for radiometers held close to the plaque or that have a large diameter.
- Secondly, while it is typical to mount irradiance sensors high on poles/masts (Section 2.2.2) and certainly above head height, measurements with a reflectance plaque are nearly always made much lower on a ship/platform for practical reasons: it is generally necessary to manipulate the radiometer (e.g., to then point to water and sky) and the plaque (e.g., to protect it when not measuring). Optical contamination from ship/platform sides, upper decks, masts, and even humans (often including those making the measurement) can be significant and difficult to quantify.

**Figure 7.**
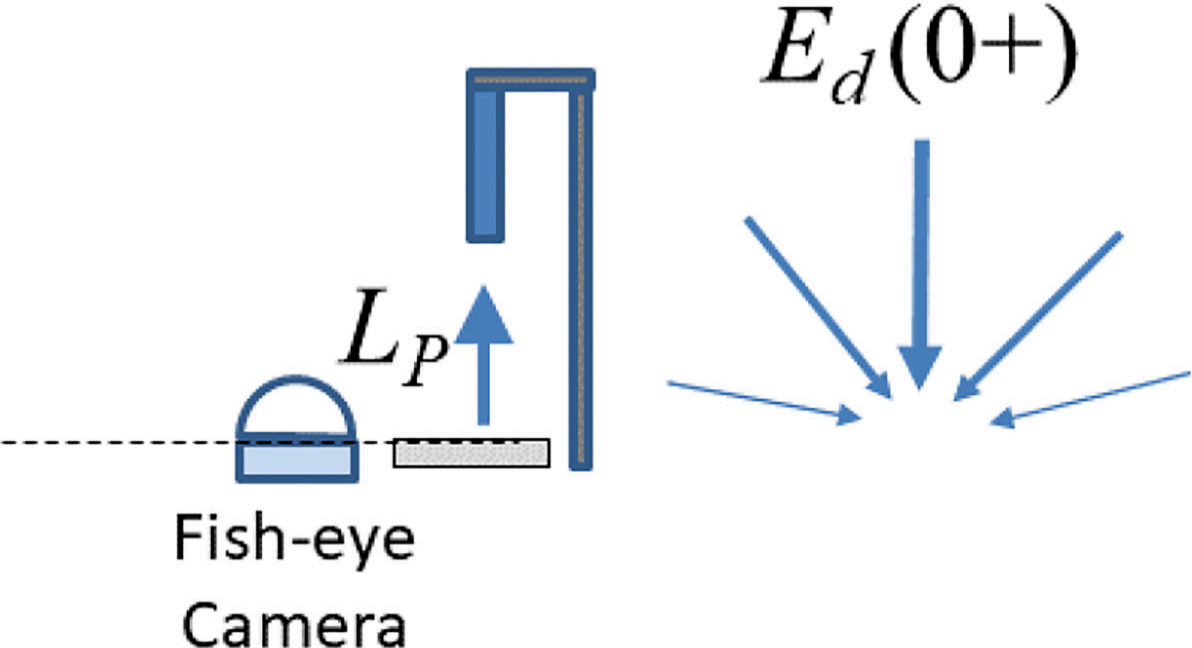
Location of fish-eye camera used for qualitative checking of shading of reflectance plaque, for comparison with Figure 4 for the direct measurement of $E_{d}^{0+}$ using an irradiance sensor, as described in Section 2.

The process of sky shading can be easily understood from fish-eye photographs taken vertically upwards at the location of a reflectance plaque – see Figure 7. Any part of the upward hemisphere that is not sky represents the optical contamination of the measurement, and this contamination will be related to the zenith cosine-weighted solid angle of sky that is replaced by the object with near-zenith objects contributing more than near-horizontal objects to the cosine integral of radiances.

Measures to estimate the uncertainties associated with shading/reflection could include experiments made with irradiance sensors, with well-characterized cosine response, located (a) alongside the plaque, and (b) on a mast above the possible optical contamination and/or experiments combining optimal and non-optimal locations [50]. Such an experiment is reported by [51] for land remote sensing applications, but the issues are clearly the same as for water remote sensing. In that study, the height of the sensor above the plaque and the position of a human observer were varied. The shading (but not reflection) effects from radiometer and observer are analyzed in detail in the model simulations of [42], for different Sun zenith angles and aerosol conditions, with the conclusion that a plaque-viewing nadir angle of 40° and relative azimuth to the Sun of 90° is recommended when viewing a ~100% reflectance plaque.

#### 3.2.5. Fouling

##### The radiometer fore-optics and the reflectance plaque should be kept clean.

When measurements made with a reflectance plaque are supervised, there should be negligible contamination of the radiance sensor fore-optics, provided that it is cleaned whenever necessary following the manufacturers’ recommendations.

Optical contamination of the plaque itself may be a significant problem because of the atmospheric deposition of particles (which may embed within the structure of some diffuser materials) of both natural and ship-related origin, marks from contact with any objects including materials used to protect the plaque during storage, etc. For example, it is recommended to keep plaques away from plastics and hydrocarbons (diesel fumes) and to build a storage box that holds the plaque fixed in a way such that the reflective surface is not in contact with anything. Obviously, humans, especially those with greasy fingers, should not touch the diffusive surface itself. The cleaning of dirty plaques is, of course, recommended, but should be accompanied by recalibration or pre/post-cleaning calibration checks.

In addition to optical contamination, plaques may change naturally from photodegradation processes related to ultraviolet exposure. For example, the reflectivity of Spectralon^™^, a proprietary form of sintered polytetrafluoroethylene (PTFE) produced by Labsphere Inc., USA, and used for both spaceborne calibration diffusers and many ground-based measurements, may change at short wavelengths because of absorption from organic impurities [52,53], which can only be removed by vacuum baking. The careful handling and storage of plaques is required to limit such degradation.

The uncertainty estimate related to fouling can be validated by comparing post-deployment calibrations before and after cleaning a plaque.

#### 3.2.6. Fast Natural Fluctuations

##### Measurements should be used only during periods of stable illumination.

Considerations and uncertainties associated with fast natural fluctuations of $E_{d}^{0+}$ over a typical measurement time scale (~1 min to 10 min) are identical to those already discussed in Section 2.2.4, except that the asynchronicity of $E_{d}^{0+}$ and $L_{w}$ measurements is inevitable for this method. In the latter context, replicate measurements, e.g., $E_{d}^{0+}$ before and after $L_{w}$, can be used.

#### 3.2.7. Bidirectional Reflectance of Plaques

##### The bidirectional reflectance of the plaque should be known.

In general, a plaque calibration is made for unidirectional illumination (typically 8°) and with hemispherical collection, using an integrating sphere, which is termed “8/h” calibration. Whereas the cosine response of irradiance sensors must be considered for the direct measurement of $E_{d}^{0+}$ (Section 2), the bidirectional reflectance of a plaque (from all illuminating directions to the single viewing direction) must be considered in the uncertainty estimate for the reflectance plaque method. This data is reported in some cases for typical white Spectralon^™^ plaques [53] and for grey Spectralon^™^ plaques [42,54], but they may be unknown for other materials, including grey cards. A full characterization of the optical properties of a plaque will include polarization sensitivity in the calibration process [55]. The full four-dimensional and reciprocal Mueller matrix bidirectional reflectance distribution function of sintered polytetrafluoroethylene is reported at four wavelengths in [56]. The uncertainty associated with the imperfect Lambertian response of a plaque can be validated by comparison, for a range of Sun zenith angles, with a zenith-pointing irradiance sensor, if the latter has a sufficiently characterized cosine response and is associated with a full uncertainty analysis.

### 3.3. Variants on the Method for Measurement of $E_{d}^{0+}$ Using a Downward-Pointing Radiance Sensor and a Reflectance Plaque

Multiple measurements can be made with different plaques [18], e.g., of different reflectivity, to reduce/validate the uncertainties associated with individual plaques (calibration, optical contamination/degradation, bidirectionality, etc.).

Although not used for the measurement of $E_{d}^{0+}$ as such, it is interesting to note the use of a “blue tile” reported by B.C. Johnson in Section 7.10 of [18]. This specially-manufactured reflectance plaque has spectral properties similar to those of blue water, and so provides an intercomparison target, which allows the testing of some aspects of above-water $L_{w}$ protocols with some aspects of radiometer characterization, such as straylight.

## 4. Estimation of $E_{d}^{0+}$ from Direct Sunphotometry and a Clear Sky Atmospheric Model

**Figure 8.**
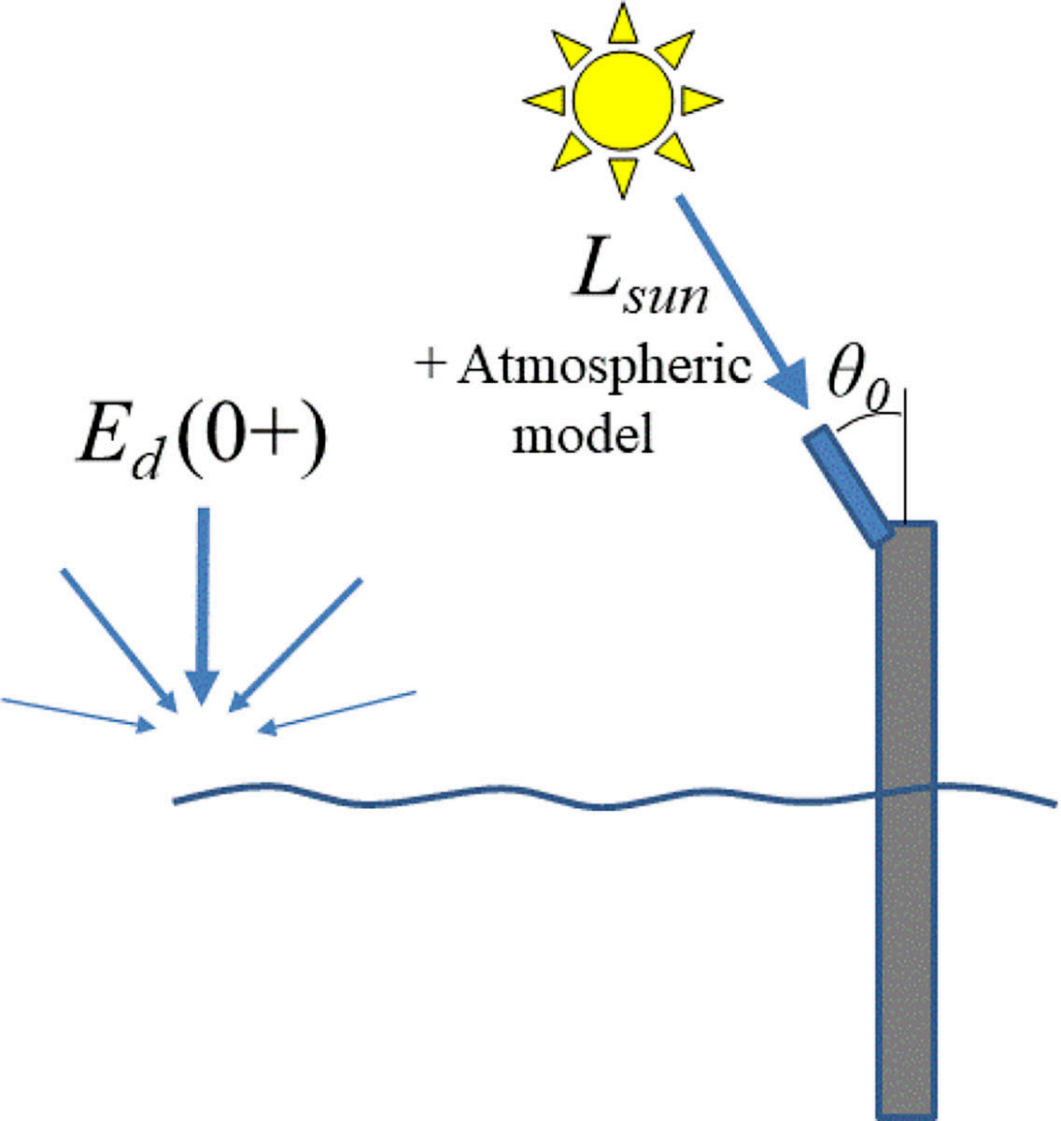
Schematic of direct Sun measurement for the estimation of $E_{d}^{0+}$.

As an alternative to the direct measurement of $E_{d}^{0+}$ using a vertically-pointing irradiance sensor as described in Section 2, it is possible to estimate aerosol optical thickness by measuring the direct Sun radiance with a sunphotometer and estimate the total atmospheric transmittance with this and other inputs—see Figure 8. This method was originally developed for satellite validation measurements using the hand-held SIMBAD(A) radiometer [57], and has the interesting feature for satellite validation studies of providing more information on atmospheric parameters than just the $E_{d}^{0+}$ measurement described in Section 2 and 3. In the hand-held SIMBAD(A) protocol, only aerosol optical thickness is measured, but for automated Sun/sky radiometers, such as those of the AERONET-OC network [26] with many other pointing scenarios, many extra atmospheric parameters can be estimated, including aerosol size distribution and phase function [58].

This method was described in the NASA Ocean Optics Protocols [46] as above-water radiometry “Method 3”, in combination with measurements of water-leaving radiance using a vertical polarizer, as implemented for the SIMBAD(A) radiometer. However, this method for estimating $E_{d}^{0+}$ may be combined with different methods for estimating $L_{w}$, e.g., above-water methods without a vertical polarizer, and so is described here as a generic method for estimating $E_{d}^{0+}$.

The pointing accuracy required for direct Sun measurements generally requires a very stable platform, such as a fixed offshore structure as in the AERONET-OC protocol [26], for unsupervised measurements, or can be achieved by a hand-held sunphotometer, e.g., SIMBAD(A) radiometer [57]. However, the feasibility of making direct Sun measurements from a moving platform has been demonstrated for an airborne radiometer [59], so it is conceivable that such measurements may be made in the future from structures with some movement, e.g., buoys..

### 4.1. Measurement Equation

The full measurement equations for this method are described in [57] using a notation typical for atmospheric radiative transfer studies, which does not explicitly mention $E_{d}^{0+}$. For compatibility with the rest of this review, these equations are rewritten here in a form that facilitates the identification of $E_{d}^{0+}$ itself.

Thus, the total (direct and diffuse) downward (Sun to water) atmospheric transmittance, $T_{0}$, is defined by:

(5) $$T_{0}=\frac{E_{d}^{0+}}{E_{d}^{TOA}}$$

and the downwelling irradiance at Top of Atmosphere, $E_{d}^{TOA}$, is estimated from:

(6) $$E_{d}^{TOA}=F_{0}cos\vartheta_{0}{\left(\frac{d_{0}}{d}\right)}^{2}$$

where $F_{0}$ is the extraterrestrial solar irradiance for mean Sun–Earth distance $d_{0}$, e.g., tabulated by [60], $\vartheta_{0}$ is the Sun zenith angle, and d is the Sun–Earth distance at the time of the measurement, which can be easily calculated from position and date/time using earth orbital models.

Combining Equations (5) and (6) gives:

(7) $$E_{d}^{0+}=T_{0}F_{0}\cos\vartheta_{0}{\left(\frac{d_{0}}{d}\right)}^{2}$$

$T_{0}$ is estimated using a clear sky radiative transfer model, e.g., [61], which takes as input vertically integrated ozone amounts (obtained from extraneous data such as Total Ozone Mapping Scanner satellite data and/or meteorological models or climatologies), $\vartheta_{0}$, surface atmospheric pressure (which influences Rayleigh optical thickness and may be obtained from simultaneous surface measurements or from appropriate meteorological models), and aerosol optical thickness, $\tau_{a}(\lambda )$—see Equation (7) of [57]. The impact of other absorbing gases and absorbing aerosols and other parameters such as surface reflectance may be included in the atmospheric radiative transfer model, if necessary.

In the estimation of $T_{0}$, the effects of multiple scattering from surface to atmosphere back to surface are generally neglected. These effects can be important over reflective waters and nearby land, especially at short wavelengths, where the spherical albedo of the atmosphere becomes large; for a more complete treatment, see [6].

The aerosol optical thickness $\tau_{a}(\lambda )$ is deduced from direct Sun measurements taking account of sunphotometer calibration, Earth–Sun distance variation $d/d_{0}$, Sun zenith angle $\vartheta_{0}$, and including corrections for molecular scattering and gaseous absorption, which is considered to be mainly due to ozone—see Section 4.1 of [57], including Equations (5) and (6). In theory, sky radiance information (in the principal plane and almucantar, especially aureole), in addition to direct sunlight measurements, could be used to better determine the aerosol type, and therefore better estimate the atmospheric transmittance. In practice, only aerosol optical thickness is used to estimate atmospheric transmittance from AERONET-OC and SIMBAD(A) measurements, because the anisotropy factor of the aerosol phase function is quite constant for most aerosol models [62]. However, when aerosols are absorbing, the impact of absorption can be significant [63].

The Ångström exponent for the spectral variation of $\tau_{a}(\lambda )$ can also be computed, and in the SIMBAD protocol it is used in the skyglint correction for $L_{w}$, but is not needed for the computation of $E_{d}^{0+}$.

The calculation of $T_{0}$ required for this $E_{d}^{0+}$ measurement protocol is comparable to the computation of $E_{d}^{0+}$ made in satellite data processing software, e.g., SeaDAS.

### 4.2. Protocol-Dependent Sources of Uncertainty

In addition to the radiometer-related sources of uncertainty that arise from imperfections in the radiometers themselves, including the Bouguer–Langley calibration, the measurement of above-water downwelling irradiance from direct Sun radiometry and atmospheric modeling has a number of sources of uncertainty relating to the measurement equation and deployment conditions. These protocol-related sources of uncertainty are described in Sections 4.2.1–4.2.6.

#### 4.2.1. Atmospheric Radiative Transfer Model

##### The atmospheric radiative transfer model and its inputs (extraterrestrial solar irradiance, absorbing gases, atmospheric pressure, Sun zenith angle, etc.) should be accurate.

The atmospheric radiative transfer model used to estimate $T_{0}$ has both intrinsic uncertainties, which are associated with models and simplifications of many complex atmospheric optical processes, as well as uncertainties in the various input parameters (aerosol parameters, absorbing gas amounts, atmospheric pressure, Sun zenith angle, etc.) and which propagate through the model. The extraterrestrial solar irradiance also includes some uncertainty; ideally, the same solar irradiance data will be used for in situ and satellite data processing.

The estimation of uncertainty from all these sources is complex and is described in detail in Section 5 of [57], except for the adjacency effect of multiple surface–atmosphere scattering, which was mentioned in Section 4.1.

An intercomparison of atmospheric radiative transfer codes and discussion of issues can be found in [64].

#### 4.2.2. Sky Conditions

##### The atmosphere should be cloud-free and horizontally homogeneous.

The atmospheric radiative transfer model used to estimate $T_{0}$ assumes that the atmosphere is horizontally homogeneous and, in particular, contains no clouds. This assumption is valid for the design conditions of clear sky satellite validation, but significant and difficult-to-estimate uncertainties will arise if this assumption is violated, e.g., for a partially cloudy sky. In the SIMBAD(A) and AERONET-OC protocols, automated quality control steps identify when the direct Sun measurement is affected by clouds or haze near the Sun, and remove such data from processing. In the SIMBAD(A) protocol, the human observer can also identify suboptimal conditions, such as clouds somewhere else in the sky, and quality flag such data accordingly.

#### 4.2.3. Pointing Effects

##### The sensor FOV should contain entirely the Sun and be centered on the Sun.

While high pointing accuracy is crucial for direct Sun measurements, this can be well achieved by both robotic and handheld systems allowing for fine pointing adjustments. The field of view of sunphotometers is by design small, e.g., 1° to 3°, and typically not much larger than the Sun’s linear angle of about 0.53°, to minimize the contribution of atmospheric scattering yet completely cover the Sun disk.

Inadequate pointing accuracy can be identified from replicate measurements and/or very high apparent optical thickness and corresponding measurements removed during quality control steps.

Uncertainties associated with direct Sun pointing may be grouped with other uncertainties in the measurement of aerosol optical thickness.

#### 4.2.4. Shading

##### The direct path from Sun to sensor should be free of obstructions.

Shading of the direct Sun measurement by the presence of solid objects is generally not a problem because—in contrast to the direct measurement of $E_{d}^{0+}$ with an irradiance sensor where the whole upward hemisphere should be free of obstructions—for direct Sun measurements, only the direct Sun path must be free of obstructions. For unsupervised measurements, most structure shading will be very obvious in direct Sun measurements, and can be automatically removed either a priori, by defining a range of acceptable viewing azimuth angles, or a posteriori, by eliminating very low radiance values. Minor obstructions such as wires and cables potentially in the field of view should be eliminated during deployment, and other occasional obstructions (birds, humans) can be monitored by video camera. For supervised measurements, any structural shading can easily be identified and avoided.

On some platforms, there may be a risk of optical contamination from atmospheric steam or smoke emissions and other exhaust gases (air conditioning, etc.).

#### 4.2.5. Fouling

##### The sensor fore-optics should be clean.

Sunphotometers are always associated with a pointing mechanism that is either robotic or human, and so can generally be protected from most fouling mechanisms when not measuring.

Nevertheless, some fouling of the fore-optics may occur for long-term unsupervised deployments because of sea spray, rain droplets, and/or spiders and insects, etc.

Major fouling events can be identified by time series analysis of data and/or video camera imagery. The uncertainty estimate related to fouling can be validated by comparing post-deployment calibrations before and after cleaning [26].

#### 4.2.6. Fast Natural Fluctuations

##### Measurements should be used only during periods of stable illumination.

This method for $E_{d}^{0+}$ can only be used in ideal clear sky conditions, where fast natural fluctuations of $E_{d}^{0+}$ do not occur. The latter can easily be detected by replicate measurements, and the corresponding measurement sequence can be eliminated.

### 4.3. Variants on the Method of Measurement of $E_{d}^{0+}$ from Direct Sunphotometry and a Clear Sky Atmospheric Model

As mentioned previously, this protocol can be used with human or robotic pointing systems. Since this protocol has very different assumptions and very different sources of uncertainty from the protocol using a vertically-pointing irradiance sensor (Section 2), there is significant added value to combine the sunphotometric estimation of $E_{d}^{0+}$ with the direct measurement of $E_{d}^{0+}$ using an irradiance sensor, as proposed in the OSPREY system [65].

## 5. Estimation of $E_{d}^{0+}$ from Underwater Measurements

It is common for underwater radiometric measurements of the profile with depth, z, of nadir upwelling radiance, $L_{un}(z)$, to be accompanied by underwater measurements of downwelling irradiance, $E_{d}(z)$. Historically, $E_{d}^{0+}$ was often estimated from these underwater measurements by extrapolation to just beneath the surface and transmission across the air–water interface. However, the temporal variability of $E_{d}(z)$ associated with wave focusing/defocusing is particularly difficult to remove, and this method for estimating $E_{d}^{0+}$ has been replaced by the direct above-water $E_{d}^{0+}$ measurement, and will not be discussed further in this review. A detailed description of protocols for measuring $E_{d}(z)$, the spectral diffuse attenuation coefficient of downwelling irradiance, $K_{d}(\lambda ,z)$, and, if considered useful, $E_{d}^{0+}$, can be found in the NASA Ocean Optics protocols [66].

Outside the satellite validation context, underwater measurements of $E_{d}(z)$ are still relevant for the estimation of optically and biologically important parameters such as $K_{d}(\lambda ,z)$, and related parameters such as euphotic depth.

## 6. Conclusions

### 6.1. Summary of the State of the Art

This paper reviews the current state of the art of protocols for the measurement of downwelling irradiance for the validation of satellite remote sensing data over water. In the FRM context, particular attention is paid to the protocol-related elements of the measurement uncertainty budget. These aspects of the protocol are discussed with reference to documented studies, and guidelines are provided on how to estimate such uncertainties, e.g., design of experiments and/or model studies.

Three basic measurement protocols have been identified:

- Direct above-water measurement of $E_{d}^{0+}$ with an upward pointing irradiance sensor
- Estimation of $E_{d}^{0+}$ using a downward pointing radiance sensor and a reflective plaque
- Estimation of $E_{d}^{0+}$ from direct sunphotometry and a clear sky atmospheric model

A fourth measurement method that was previously used, estimating $E_{d}^{0+}$ from the underwater vertical profiles of $E_{d}(z)$, is now considered inappropriate, and is no longer recommended. This method remains relevant for the measurement of $E_{d}(z)$ and related parameters such as diffuse attenuation coefficient, but not $E_{d}^{0+}$.

The main body of this paper is summarized in Table 1, which lists the equipment needed, method variants, and any special issues, and in Table 2. The latter summarizes the components of the uncertainty estimation giving ideal conditions, recommendations for best practice, and approaches to estimating uncertainty, but excludes any uncertainties arising from radiometer imperfections, such as calibration, thermal sensitivity, spectral response (straylight/out of band effects), non-linearity, and angular (cosine) response.

For the “irradiance sensor” and the “reflectance plaque” methods, the main challenge is to deploy the radiometer/plaque sufficiently high enough to avoid any shading. In this context, “shading” does not only refer to the obvious shadowing of direct Sun, but also refers to the difference between the unobstructed hemisphere of Sun and sky radiance and the reality of measuring in situations where the radiometer/plaque are not higher than all the other structures. For the “irradiance sensor” method, it is also a major challenge to have a sensor that is sufficiently well-designed and well-characterized as regards angular (cosine) response [28].

### 6.2. Irradiance Sensor or Reflectance Plaque?

The preparation of this review stimulated considerable discussion within the community on the pros/cons of the reflectance plaque method as compared to the irradiance sensor method in addition to the question of whether the reflectance plaque method radiance sensor needs to be calibrated (see Section 3.1.1). When correctly applied, the reflectance plaque method can clearly meet the criteria expected of an FRM. However, in practice, this method has often been associated with less rigorous implementation. Specifically, recognizing that the reflectance plaque is performing the same function as the fore-optics of an irradiance sensor, which collects light from the upward hemisphere according to a zenith cosine weighting and directs that light to a photodetector, it is necessary that:

1. There be no humans above the level of the reflectance plaque/irradiance sensor (and thereby affecting the sky radiance contributing to $E_{d}^{0+}$ in a way that is highly variable and essentially not quantifiable in an uncertainty estimate),
2. The reflectance plaque/irradiance sensor be mounted as high as possible on the ship/platform, typically higher than any superstructure elements with significant solid angle as viewed from the plaque/sensor,
3. The reflectance plaque/irradiance sensor be mounted on a fixed structure, not hand-held, and associated with an inclinometer allowing the estimation of uncertainties associated with non-horizontal/vertical measurements,
4. The measurements made using the reflective plaque/irradiance sensor be supported by experiments and/or simulations to estimate the measurement uncertainties associated with any superstructure shading of the plaque/irradiance sensor.

### 6.3. Future Perspectives

In contrast to the more difficult $L_{w}$ measurement, where there has been considerable evolution and diversity since the publication of the NASA Ocean Optics Protocols [20], measurement protocols for $E_{d}^{0+}$ seem now to be quite mature and stable.

Future improvements to $E_{d}^{0+}$ measurements are expected to come from the following developments:

- Improvements in the design and usage of calibration monitoring devices, which can be used in the field, are likely to improve the identification of fore-optics fouling and radiometer sensitivity changes.
- Model simulations of the 3D light field and experiments for deployments with structures above the irradiance sensor/reflectance plaque are likely to improve estimations of related uncertainties.
- Improvements in the stability and reduction in the cost of telescopic masts may reduce superstructure shading effects.
- Reduction in the cost of pointing systems, thanks to the video camera surveillance industry, should improve the protection (“parking”) of irradiance sensors when not in use, and thus reduce fouling for long-term deployments.
- Improvements in automatic gimballing systems might reduce the tilt effects for the irradiance sensor method.
- Greater use of full sky imaging cameras, whether calibrated (expensive) or not (inexpensive), will allow the better identification of suboptimal measurement conditions.

As regards the future for the validation of water reflectance more generally:

- The tendency to move to highly automated systems with long-term, e.g., one year, essentially maintenance-free deployments is likely to significantly improve the quantity of data available for validation.
- The advent of operational satellite missions such as NPP/VIIRS, Sentinel-3/OLCI, Sentinel-2/MSI, and Landsat-8/OLI with the need for a guaranteed long-term validated data stream will increase the need for FRM.
- The huge increase in optical satellite missions used for aquatic remote sensing will also increase the need for highly automated measurement systems.
- As regards the needs of the validation community, it is recommended to:
- Update this review, e.g., on a 10-year time frame, to take account of developments in the protocols, particularly in the estimation of uncertainties. Such an update is best preceded by community discussion at an international workshop.
- Organize regular, e.g., on a two-year time frame, intercomparison exercises to ensure that measurement protocols and scientists remain state of the art (as required by the FRM context).

Although not targeted by this review, it is possible that the considerations developed here may be useful for other applications where $E_{d}^{0+}$ measurements are needed, including the validation of satellite-derived photosynthetically available radiation products [67], the validation of surface reflectance over land, and the monitoring of solar irradiance for the solar energy industry, for agriculture, for the building industry, for the estimation of the Earth’s radiation budget, and absorbing atmospheric gases, etc.

## Supplementary Material

xml

## Figures and Tables

**Table 1.** Summary of the three measurement methods as regards equipment, method variants, and special issues.

	Upward-Pointing Irradiance Sensor	Radiance Sensor and Reflective Plaque	Direct Sunphotometry

Equipment	Irradiance sensor (cosine response) Inclinometer	Radiance sensor Reflective plaque Inclinometer	Sunphotometer (radiance) sensor Pointing mechanism Atmosphere radiative transfer model

Variants	Surfacing of underwater drifting floats. Shadowband for diffuse/direct.	White/grey plaques	Hand-held or robotic pointing

Other notes		Uncalibrated radiometers? (see Section 3.1.1) Plaque viewing nadir angle? (see Section 3.1.2)	

**Table 2.** Summary of the three measurement methods, including components that must be considered for the uncertainty estimation.

Method	Upward-Pointing Irradiance Sensor	Radiance Sensor and Reflective Plaque	Direct Sunphotometry

Plaque cal and characterization	N/A	I: BRDF-calibrated, homogeneous plaque fills FOV R: Tests to check FOV U: Plaque certificate including BRDF, experiments for homogeneity and height above plaque/FOV	N/A

Tilt/pointing	I: Deploy vertical R: Monitor with inclinometer U: Modeling/experiments	I: Deploy horizontal R: Monitor with inclinometer U: Modeling/experiments	I: Sensor FOV contains and centered on Sun R: Small FOV, accurate pointing, check AOT U: Via estimation of AOT

Superstructure shading	I: Deploy above all structures R: Use mast and fish-eye photos U: Experiments (different heights/locations) and modeling	I: Deploy above all structures (except radiometer) R: Use mast and fish-eye photos U: Experiments (different heights/locations) and modeling	I: Clear radiometer–direct Sun path R: Check with video surveillance and data QC U: N/A (if not rejected)

Fouling	I: Keep fore-optics clean R: Inspect/clean/protect, monitor with portable cal devices U: Pre-/post-cleaning cal of radiometer	I: Keep radiometer fore-optics and plaque clean R: Inspect/clean/protect, monitor radiometer with portable cal devices U: Pre-/post-cleaning cals for radiometer and plaque	I: Keep fore-optics clean R: Inspect/clean/protect U: Pre-/post-cleaning cals

Fast natural fluctuations	I: Reject if unstable illumination R: Compare replicates/time series U: S.D. of accepted measurements	I: Reject if unstable illumination R: Compare replicates/time series U: S.D. of accepted measurements	I: Reject if unstable illumination R: Compare replicates/time series U: S.D. of accepted measurements

Sky conditions and atmospheric r/t model	N/A	N/A	I: Perfectly cloud-free sky, horizontally homogeneous atmosphere and surface. Perfect r/t model and inputs R: Reject if clouds detected. Intercompare r/t models, check inputs U: Modeling. See Section 4.2.1

BRDF: bidirectional reflectance distribution functions; I = Ideal conditions; R = Recommendations; U = Uncertainty estimation; Cal = calibration; FOV = field of view; AOT = aerosol optical thickness; r/t = radiative transfer; S.D. = standard deviation; N/A = Not Applicable. See text for more details on each topic.
